# Translation, Cross-Cultural Adaptation, and Validation of the Storm Fear Questionnaire in Brazilian Pregnant Women Exposed to an Extreme Climate Event

**DOI:** 10.3390/brainsci16030288

**Published:** 2026-03-04

**Authors:** Miguel G. Garcia, Bernardo B. C. Baldi, Pedro Giuberti, João Henrique Chrusciel, Sofia T. Berlaver, Gabriela C. Machado, Martina A. Lodi, Christian H. Kristensen, Saulo Gantes Tractenberg, Rodrigo Grassi-Oliveira, Thiago W. Viola

**Affiliations:** 1 Developmental Cognitive Neuroscience Lab (DCNL), Pontifical Catholic University of Rio Grande do Sul (PUCRS), Porto Alegre 90619-900, Brazil; miguelgomesgarcia@gmail.com (M.G.G.); bernardobaldi@gmail.com (B.B.C.B.); giubertipedro@gmail.com (P.G.); chruscieljoao@gmail.com (J.H.C.); stberlaver@gmail.com (S.T.B.); gabiclossmachado@gmail.com (G.C.M.); martilodi13@gmail.com (M.A.L.); rodrigo.grassi@pucrs.br (R.G.-O.); 2 Department of Psychology, Universidade do Vale do Rio dos Sinos (UNISINOS), São Leopoldo 93022-750, Brazil; saulogantes@gmail.com; 3 Centre for Studies and Research in Traumatic Stress (NEPTE), Pontifical Catholic University of Rio Grande do Sul (PUCRS), Porto Alegre 90619-900, Brazil; christian.kristensen@pucrs.br; 4 Translational Neuropsychiatry Unit, Department of Clinical Medicine, Aarhus University, 8200 Aarhus, Denmark

**Keywords:** Storm Fear Questionnaire, fear of storms, extreme weather, artificial intelligence, validation study, Brazil

## Abstract

**Highlights:**

**What are the main findings?**

- The Brazilian version of the SFQ showed excellent acceptability and high internal consistency in a sample of 268 postpartum women exposed to an extreme storm-related event.
- SFQ total scores correlated strongly with posttraumatic stress symptoms and moderately with depressive symptoms, supporting evidence of convergent validity.

**What are the implications of the main findings?**

- There is a need to assess fear of storms in populations exposed to extreme weather events and their mental health outcomes, given the increasing occurrence of events related to these phenomena.
- The Brazilian version of the SFQ enables standardized screening and monitoring of fear of storms in a context recently impacted by a large-scale flood.
- The Brazilian version of the SFQ supports the use of a reliable total score to assess the severity of fear of storms and to differentiate known groups based on flood exposure.
- The instrument may facilitate clinical screening and research on fear of storms and disaster mental health in Brazil.

**Abstract:**

**Background:** Extreme weather events, such as storms, may evoke intense fear in individuals and impair their daily functioning, resulting in significant distress. In Brazil, recent climate-related disasters have highlighted the need to assess storm fear in the population. **Objective:** This study aimed to translate, adapt, and validate the Storm Fear Questionnaire (SFQ) for the Brazilian context. **Methods:** Translation and adaptation were conducted, followed by back-translation, review by an expert panel, and acceptability assessment by the target population. For the psychometric evaluation, a sample of 268 postpartum women exposed to a flood in southern Brazil completed the SFQ and the following questionnaires: the Posttraumatic Stress Disorder Checklist for DSM-5 (PCL-5), Beck Depression Inventory II (BDI-II), and the Pregnancy Experience Scale—Brief Version (PES-Brief). **Results:** The instrument showed excellent acceptability in the target population and good content validity. Regarding criterion validity, Pearson correlations indicated high convergence between the SFQ and PCL-5 and moderate convergence with the BDI-II. Regarding construct validity, SFQ scores were significantly higher among postpartum women who had to leave their homes due to the flood or had their houses affected by floodwaters. The first factor generated in the factor analysis explained 35.2% of the variance, with 14 out of 15 items presenting loadings greater than 0.40. Internal consistency was high (α = 0.88). **Conclusions:** The Brazilian version of the SFQ is a valid and reliable instrument for assessing fear of storms. Future studies are needed to evaluate the instrument’s applicability in diverse populations across the country.

## 1. Introduction

Extreme weather events have become increasingly frequent and intense in recent decades, driven by global climate change [1]. In Brazil, this phenomenon has manifested as an increase in severe storms and cyclones, as well as the risk of floods and intense winds with high destructive potential, posing a threat to the lives of affected individuals [1]. In May 2024, the state of Rio Grande do Sul experienced the largest natural disaster in its history, with large-scale floods affecting more than 2.4 million people across 478 municipalities [2]. Porto Alegre, the state capital, had approximately 30% of its urban territory flooded, with more than 160,000 people directly impacted, marking the rainiest month in the city’s history [3].

Given this scenario, several questions have been raised regarding flood containment and prevention infrastructure, as well as population protection and physical healthcare for people affected by floods [4]. However, the impact on mental health remains an emerging topic in discussions about the consequences of floods [5,6,7]. Thus, there is a need to understand the psychological effects of these disasters, especially the development of specific fears, such as fear of storms, to improve assessment and mental healthcare for the population [5,6,8].

Storm phobia is characterized by excessive and persistent fears related to severe weather conditions and is frequently associated with anxiety symptoms, avoidance behaviors, and significant emotional distress [5,6,7]. According to the Diagnostic and Statistical Manual of Mental Disorders (DSM-5-TR), it is classified as a specific phobia of the natural environment, alongside other phobias such as fear of water or heights [9]. Symptoms include anxious anticipation even days before the storm, avoidance behaviors (such as failing to attend appointments), physiological responses, and excessive monitoring of weather forecasts [5,7]. Westefeld [8], in an exploratory study with 81 North American individuals who reported fear of storms, described significant impacts on participants’ daily functioning, whether at home or at work, or in avoiding leaving home when rain was forecasted. Although few participants sought psychological treatment, the reports indicated intense symptoms, suggesting possible underdiagnosis.

From this perspective, the literature describing the phenomenology of fear of storms and its treatment is limited, focusing primarily on psychological symptomatology after adverse events and devoting little attention to a significant pre-existing fear of storms [5,7,8]. Furthermore, storm phobia is often underreported, as many individuals feel ashamed of their symptoms and avoid seeking treatment [7]. Stinson and colleagues [10] estimated that approximately 2% of the population in the United States and Canada will experience storm phobia. However, prevalence data are not available for the Brazilian context, reflecting the scarcity of studies on this topic.

To date, no instrument has been adapted and validated for the Brazilian context that assesses, independently and directly, fear of storms as a specific phobia. Although the international literature presents initiatives to measure fear of storms, there is still no Portuguese-language version validated for the national context [7,11]. Therefore, an important limitation remains in identifying and assessing the severity of suffering related to anxiety or trauma associated with storms, both in clinical settings and in population-based research, such that assessment generally relies on a clinical interview to determine whether the DSM-5-TR diagnostic criteria are met [7,9].

In view of this need, the Storm Fear Questionnaire (SFQ), developed by Nelson and colleagues [7], is a 15-item instrument answered on a 5-point Likert-type scale to measure the severity of fear of storms in adults. Factor analysis identified a unidimensional structure with excellent internal consistency (α = 0.95) in a sample of Canadian university students, as well as evidence of convergent and discriminant validity with measures of anxiety and general worry. In addition to its original version, the SFQ has also been adapted and validated in other countries, such as Turkey, where it retained its factor structure and showed excellent internal consistency (α = 0.943) in a sample of adults from the general population [11]. These findings demonstrate its psychometric robustness and cross-cultural applicability, reinforcing the importance of adapting it to the Brazilian context.

Given the growing relevance of this topic due to the increasing occurrence of extreme weather events, the present study aimed to translate and cross-culturally adapt the SFQ and to gather psychometric evidence of its reliability and validity in the Brazilian context (i.e., content, criterion, and construct validity), thereby contributing to the availability of valid and reliable mental health assessment tools for use in the context of extreme weather events.

## 2. Materials and Methods

### 2.1. Translation and Adaptation

The translation and cross-cultural adaptation of the SFQ for the Brazilian context were conducted in accordance with the test translation and adaptation guidelines proposed by the International Test Commission [12], which guides the process to ensure linguistic, cultural, functional, and psychometric equivalence of adapted instruments across different contexts. To this end, five stages were carried out to achieve semantic and conceptual equivalence in the adaptation of the instrument, including procedures related to the use of artificial intelligence (AI) as an auxiliary tool in the initial translation, following recommendations in the recent literature [13,14].

AI, particularly large language models, has been used as an initial translation aid based on studies that recognize the accuracy of its translations and its potential when combined with human review and validation [13,14,15,16,17,18]. The first stage consisted of translating the original version of the instrument in English into Brazilian Portuguese with the assistance of the large language model (LLM) GPT-4o [19], configured with a low temperature (exact prompts used are available in the Supplementary Material). This setting controlled the randomness of the model’s word choice, whereby higher values increased diversity and creativity at the expense of coherence, whereas lower values, used in the translation of the SFQ, promoted more accurate and consistent translations by minimizing interpretive variation [14]. The low-temperature setting also favors greater precision in replicating the prompt employed. In this regard, we repeated the translation request five times, and across all runs, GPT-4o produced very similar results, indicating stability in the generated output while underscoring the indispensability of human review.

In the second stage, in line with recommendations for human review after AI-based translation, the version produced was reviewed by two members of the research team, fluent in English and with prior experience in academic research focused on psychometrics and trauma, who made the necessary adjustments to ensure clarity, naturalness, and fidelity to the original version [13]. In the third stage, a bilingual clinical psychologist with no prior knowledge of the original instrument conducted back-translation following established back-translation guidelines, enabling a further stage of human review, with additional adjustments when necessary [13,14,20].

In the fourth stage, the revised version was submitted for evaluation by an expert panel composed of two psychologists with clinical and academic experience in psychological trauma. The specialists qualitatively analyzed the 15 items of the instrument, considering criteria such as verbal clarity, cultural appropriateness, and relevance to the construct assessed, suggesting language modifications when deemed appropriate [20]. The suggestions received were analyzed by the study authors to define the final version of the items, adapting them to the Brazilian context while respecting the original content of the instrument.

In the fifth stage, the final version, consisting of 15 items answered on a 5-point Likert-type scale, was administered to a sample of postpartum women (*n* = 10) to assess the acceptability and comprehension of the instrument’s items using a Verbal Rating Scale (VRS) [21]. For all items, the following guiding question was asked: “Did you understand what you were asked?” Responses ranged from 0 (I did not understand anything) to 4 (I understood and had no doubts).

Content validity (i.e., comprehensibility of items) was evaluated for each item. The item-level Content Validity Index (I-CVI) was calculated as the proportion of ratings falling in the “clear” range (e.g., scores of 3 or 4) divided by the total number of raters. The final version was sent to the author of the original SFQ study and was approved.

### 2.2. Sample for the Psychometric Validation Study

The sample consisted of postpartum women housed in the obstetric unit of two hospitals in Porto Alegre, Rio Grande do Sul (RS). In total, 268 postpartum women completed the questionnaires. The inclusion criteria were living in the metropolitan region of Porto Alegre during the flood period that affected the region in May 2024. Participants were required to be 18 years or older and no older than 45 years. There was no exclusion criterion related to clinical conditions. The only exclusion applied was when a participant could not complete the interview/questionnaires due to difficulty or confusion at the time of assessment (precluding valid data collection and consent). The descriptive data for the sample are presented in Table 1.

### 2.3. Instruments

In addition to the Brazilian version of the SFQ, three other instruments previously validated for use in Brazil were administered to broaden the understanding of the psychometric findings and to provide additional validity evidence. A sociodemographic questionnaire was also administered to characterize the sample, as well as a climate-disaster impact questionnaire to examine the degree of exposure to and repercussions of the flood. For operational purposes, participants were considered directly affected if they had to leave their residence for at least one week and/or reported severe residential damage.

#### 2.3.1. Posttraumatic Stress Disorder Checklist for DSM-5 (PCL-5)

The PCL-5 [22] is a self-report instrument consisting of 20 items that assess the presence and severity of symptoms related to Posttraumatic Stress Disorder (PTSD), based on the DSM-5 diagnostic criteria. The version used in this study corresponds to the Brazilian adaptation by Lima and colleagues [23]. Items are rated on a 5-point Likert scale ranging from 0 (“not at all”) to 4 (“extremely”), yielding total scores from 0 to 80.

#### 2.3.2. Beck Depression Inventory II (BDI-II)

The BDI-II [24] is a 21-item instrument that assesses the presence and severity of depressive symptoms occurring in the two weeks before administration. The version used in this study corresponds to the Brazilian adaptation by Gomes-Oliveira and colleagues [25]. Each item is answered on an ordinal scale from 0 to 3, corresponding to statements regarding each topic assessed in the questionnaire. The total score ranges from 0 to 63, with higher scores indicating greater severity of depressive symptoms.

#### 2.3.3. Pregnancy Experience Scale—Brief Version (PES-Brief)

The PES-Brief [26] was developed to assess the subjective experience of pregnancy from both positive and negative perspectives. The scale consists of 20 items, equally divided into two factors: positive experiences (e.g., “Feelings about being pregnant at this time,” “Conversations with your husband/partner about baby names,” and “Thinking about the baby’s appearance”) and negative experiences (e.g., “Physical intimacy,” “Changes in your body caused by pregnancy,” and “Concerns about physical symptoms, such as pain or bleeding”), rated on a 4-point scale from 0 to 3. The instrument allows for the calculation of scores for each dimension or a ratio between positive and negative aspects of pregnancy and is useful for measuring psychosocial stress during the gestational period. The Brazilian version of the PES-Brief, adapted by Ferreira and colleagues [27], was used in this study to evaluate the extent to which the severity of fear of storms was related to positive and negative pregnancy experiences in a sample that witnessed and/or was affected by a climate disaster.

### 2.4. Data Collection Procedures

The study was approved by the Research Ethics Committee of the Pontifical Catholic University of Rio Grande do Sul (PUCRS) and by the committees of two hospitals (CAAE: 81250724.6.1001.5336). Postpartum women hospitalized for delivery at a public hospital in Porto Alegre were selected because they constitute a high-risk population in climate disaster scenarios, with greater vulnerability to interruptions in prenatal care, access barriers, and intensified psychosocial stress—conditions associated with adverse obstetric and mental health outcomes [28,29]. National and international evidence indicates an increase in depressive, anxiety, and posttraumatic stress symptoms in the context of extreme events [30,31]. The focus on postpartum women aimed to capture, at a clinically homogeneous and logistically accessible moment, the immediate expression of these repercussions and their link to obstetric outcomes, thereby increasing the study’s clinical relevance and external validity [28,29,32].

Data were collected at two time points. Upon admission for delivery, potential participants were approached, received explanations regarding the study’s objectives and procedures, and had the Informed Consent Form read in full. If they agreed, their consent and signatures were recorded. Next, the medical records were reviewed to extract obstetric data. After childbirth, with assisted reading to ensure comprehension and standardization, the following instruments were administered in this order: the sociodemographic questionnaire, climate-disaster impact questionnaire, PCL-5, SFQ, PES-Brief, and BDI-II. All administrations occurred in a private setting in a single session, following a previously standardized and trained operational protocol.

### 2.5. Data Analysis

Initially, descriptive statistics were conducted to characterize the sample in terms of sociodemographic and clinical variables. Criterion validity was examined using Pearson correlations between the SFQ total score and the PCL-5, BDI-II, and PES-Brief scores (negative and positive pregnancy experience subscales) to investigate evidence of convergent and divergent validity.

For construct validity, the known-groups technique was used to compare mean SFQ scores and the other scale scores between postpartum women directly affected by the flood and those not affected. These comparisons were performed using Student’s *t*-test for independent samples, and effect sizes were estimated using Cohen’s d. In addition, we examined the correlation between the SFQ total score and the duration of time participants had to leave their homes. Hierarchical linear regression models were tested with the SFQ total score as the dependent variable and the scores of the other scales that showed a significant correlation with the SFQ as potential predictors. Variables were retained or removed from the models based on their statistical significance and contribution to the explained variance of the outcome.

The instrument’s internal structure was investigated using exploratory factor analysis (EFA), with principal axis factoring as the extraction method and orthogonal (Quartimax) rotation, after verifying data adequacy using the Kaiser–Meyer–Olkin (KMO) index and Bartlett’s test of sphericity. Factor retention considered eigenvalues greater than 1, the explained variance, and item factor loadings, with 0.40 as the minimum loading criterion.

The internal consistency of the Brazilian version of the SFQ was estimated using Cronbach’s alpha coefficient, complemented by an analysis of inter-item correlations. For all inferential analyses, a significance level of 5% was adopted (*p* < 0.05). Analyses were performed using SPSS version 20.

## 3. Results

### 3.1. Content Validity

The content validity of the Brazilian version of the SFQ (Supplementary Material S1) was qualitatively verified by an expert panel, which made the necessary adjustments for conceptual and cultural appropriateness. We made minor wording adjustments to improve naturalness and conceptual alignment (e.g., Item 3 refining “storm is coming” to “storm is approaching” in Portuguese and Item 5 adapting “possible harm” to “possible dangers” to better capture the intended meaning in Brazilian Portuguese). Overall, no substantial conceptual changes were required; modifications primarily improved linguistic clarity and cultural appropriateness while maintaining fidelity to the original item content.

Subsequently, the version showed good acceptability regarding item comprehension (Table 1) when presented to an additional sample of the target population (*n* = 10), with a CVI of 0.8 or higher for all items, indicating that the translated instrument was comprehensible to the target population.

### 3.2. Criterion Validity

Regarding psychometric validity (criterion, construct, and reliability), the study was conducted with a sample of 268 postpartum women with a mean age of 27.67 ± 6.15 years. Participants’ sociodemographic data are shown in Table 2. There were no differences between the affected (*n* = 63; those who had to leave home for at least one week or reported severe residential damage) and non-affected women with respect to age, income, number of pregnancies, and educational level. 

For criterion validity, correlations were calculated between the SFQ total score and the PCL-5, BDI-II, and PES-Brief scores (Table 3). The results showed significant correlations with the PCL-5 (r = 0.616, *p* < 0.001), BDI-II (r = 0.427, *p* < 0.001), and PES-Brief negative pregnancy experience subscale (r = 0.267, *p* < 0.001), indicating high convergent validity with posttraumatic stress symptoms and moderate convergent validity with depressive symptoms and adverse pregnancy experiences. Divergent validity was indicated by the absence of a significant correlation with the PES-Brief Positive Pregnancy Experience subscale (r = 0.104, *p* = 0.093).

### 3.3. Construct Validity

Initially, construct validity was examined using the known-groups technique, comparing SFQ scores and the other scale scores between the group of participants directly affected by the flood and the non-affected group. Student’s *t*-test for independent samples was used, through which statistically significant differences were observed (t(266) = −2.32; *p* = 0.021) in the mean SFQ scores between groups (Table 4), with a higher mean in the affected group (M = 25.52; SD = 14.80) compared to the non-affected group (M = 20.85; SD = 13.60; *p* = 0.021). Differences were also found in the PCL-5 total scores, with the affected group presenting more posttraumatic symptoms than the non-affected group (*p* < 0.001).

Beyond the binary group comparison, we tested the association between SFQ total score and displacement duration, operationalized as an ordinal variable (0 = no displacement; 1 = <1 week; 2 = 1–2 weeks; 3 = 2–4 weeks; 4 = 1–2 months; 5 = >2 months). The association was statistically significant (r = 0.130, *p* = 0.034).

In the second step, a hierarchical linear regression model was fitted with the SFQ as the outcome and the PCL-5, BDI-II, and PES-Brief (negative experiences) as potential predictors, given that these instruments showed significant correlations with the SFQ. The binary variable affected versus non-affected was also included as an additional predictor, considering that the SFQ scores differed significantly between these groups. Only one final significant model was obtained, including exclusively the PCL-5 as a strong predictor of the SFQ (R^2^ = 0.37; F = 156.6; *p* < 0.0001), with the remaining variables excluded because they did not contribute significantly to the model fit. This model explained 37% of the variance in the SFQ, with the coefficient indicating that for each one-point increase in the PCL-5 score, there was an estimated increase of 0.66 points in the SFQ (standard error = 0.05).

In the third step, the factor structure of the SFQ was tested. The KMO index (0.916) and Bartlett’s test of sphericity (*p* < 0.001) indicated that the data were adequate for EFA. The EFA indicated a single-factor solution (eigenvalue > 1). The first factor (eigenvalue = 5.28) accounted for 35.2% of the total variance. Almost all items loaded on this factor, and no additional factor presented an eigenvalue exceeding 1. Except for the last item, all other items showed loads greater than 0.50 on this first and primary factor (Table 5).

### 3.4. Internal Consistency

The SFQ score was 21.9 ± 13.9 in the overall sample. Table 6 presents the correlation coefficients, as well as the means and standard deviations of the SFQ items in the sample, indicating significant correlations among all items of the instrument, except for the last item, which correlated only with 6 of the remaining 14 items. The instrument’s internal consistency was excellent (α = 0.88) for the Brazilian version of the SFQ in the analyzed sample, suggesting that the items coherently assessed the same psychological construct.

We additionally calculated Cronbach’s alpha with Item 15 removed because it did not meet the loading criterion in the EFA. Internal consistency increased slightly (α = 0.89).

## 4. Discussion

This study aimed to translate and adapt the SFQ for Brazil and to seek evidence as to whether this instrument could be a valid and reliable psychometric tool to measure the degree of severity of fear of storms in a Portuguese-speaking sample that experienced an extreme weather event. Translation and cross-cultural adaptation were carried out following the steps recommended by the ITC [12], using AI as an auxiliary tool in the translation process, optimizing research time and costs, in line with the literature that consistently recommends concomitant human review [13,14]. This resulted in a version that was appropriate and well understood by the target population. Back-translation confirmed that the translated version preserved the original meaning. The factor structure, internal consistency, criterion validity, convergent and divergent validity, and complementary between-group analyses were evaluated, demonstrating significant psychometric results. In addition to the original study by Nelson and colleagues [7], which developed the SFQ in English, only one study was identified that adapted and validated it for another context, conducted in Turkey [11]. Moreover, it is noteworthy that the present study is the first to validate the SFQ using a sample exposed to an extreme weather event, contributing to a more precise analysis of the observed phenomena, considering the mental health impact on the population investigated.

For the Brazilian version, an exploratory factor analysis based on principal components was conducted to examine the robustness of the instrument for deriving a total score, according to the methods used in previous SFQ validation studies [7,11]. The factor structure indicated a single-factor model with eigenvalues > 1. In the first and strongest factor, almost all items exceeded the minimum value of 0.40, suggesting that the unidimensional model could yield a total score for measuring the observed construct. These results are consistent with those of studies conducted in Canada and Turkey.

Regarding Item 15, the only item that did not reach the loading threshold in the EFA, this item assesses the use of alcohol or other substances during a storm to relieve anxiety. Item 15 also showed the lowest mean score (Table 6), indicating that most participants endorsed this item at very low levels. This limited variability likely contributed to its weaker psychometric performance in the factor analysis. We nonetheless retained Item 15 for conceptual reasons. Substance use as a coping strategy is clinically relevant in stress- and disaster-related contexts, and maintaining the full item set supports content coverage and comparability with prior validations. At the same time, we agree that endorsement patterns and the meaning of using substances to cope may differ across cultural contexts and populations. In particular, in a postpartum sample, social desirability concerns, stigma, and the clinical/perinatal context (e.g., breastfeeding, medical guidance to avoid alcohol/drugs) may reduce endorsement and alter item interpretation relative to other settings (e.g., the original validation context). Although it showed weak psychometric performance in our sample, we considered it clinically relevant because substance use may represent a maladaptive coping strategy in the context of storm-related distress. This finding is aligned with the result reported by Parlak-Somuncu [11], who removed the item from the final version of the instrument for Turkey. However, in the Brazilian version, we retained Item 15 due to its potential clinical utility, both as an indicator of maladaptive coping and as a screening prompt for possible substance misuse in situations involving more severe storms.

Continuing with the construct validation steps, the version proved capable of differentiating known groups within the sample, demonstrating the instrument’s sensitivity in assessing populations exposed to extreme weather events. In addition, the PCL-5 emerged as a strong predictor of the SFQ, suggesting that measurements of posttraumatic stress symptoms and fear of storms within a sample exposed to a climate disaster may predict negative outcomes in this population.

For criterion validity, the SFQ showed significantly high correlations with posttraumatic stress symptoms and moderate correlations with depressive symptoms, yielding new evidence of convergent validity when compared to previous studies by Nelson and colleagues [7] and Parlak-Somuncu [11]. Divergent validity was assessed by comparing the SFQ with the PES-Brief Positive Experiences subscale, indicating that positive pregnancy experiences do not correlate with symptoms of specific fear of storms. Furthermore, the Brazilian version of the SFQ showed excellent internal consistency in the postpartum sample, supporting the instrument’s reliability across different contexts for assessing the same construct, as in previous studies.

Regarding the translation process, the GPT-4o model was used following recommendations in the recent literature, which indicate that the resource produces good results when adopting a hybrid model of AI-assisted translation and human review [13,14]. To this end, a low-temperature prompt setting was used, resulting in a translation faithful to the original content, while allowing context-appropriate changes by human reviewers. With the low-temperature setting, repeating the same translation prompt can yield outputs that are highly similar or even identical across runs on different machines. Together with the human review and qualitative analysis performed by the study authors and expert panel, the translated instrument demonstrated good acceptability in the target population, achieving satisfactory scores for all items on the content validity index. These findings reinforce the development of the scope for AI use in academic research ethically and responsibly as an auxiliary tool aimed at achieving a better cost–benefit ratio and time optimization in scientific production [14]. However, regarding the use of AI and potential biases of AI-assisted translation, these include the risk of overly literal phrasing and the possibility of missing cultural nuances. To mitigate these risks, we explicitly relied on expert review and consensus-based adjudication, using back-translation as a verification step. Discrepancies were resolved through discussion among the research team and expert reviewers, prioritizing semantic and conceptual equivalence (rather than literal word-for-word matching), and ensuring clarity and naturalness in Brazilian Portuguese.

In this sense, the SFQ appears promising for screening, monitoring, and planning psychological interventions in the context of recent climate disasters. The instrument showed good acceptability for use by mental health teams to identify individuals with greater emotional vulnerability in the face of extreme weather events, thereby optimizing mental healthcare in emergency contexts. It proved to be a psychometrically robust instrument with broad applicability in the current scenario of forecasts of new extreme weather events, presenting itself as a reliable tool for screening the specific fear of storms in the Brazilian context.

The study’s limitations include the use of a sample composed only of postpartum women, which restricts the generalizability of the findings. Future studies should evaluate measurement invariance across regions and demographic subgroups and test the SFQ in non-perinatal and male samples. Also, the small sample size (*n* = 10) for the pre-test and the content validity of the SFQ were minor limitations. In addition, the cross-sectional nature of the study precludes causal inferences and conclusions regarding symptom persistence over time. The cross-sectional design limits the psychometric evidence that can be provided. Specifically, the current data did not allow assessment of test–retest reliability or stability over time, nor do they support temporal measurement invariance testing. A confirmatory factor analysis (CFA) was not conducted; given the available sample size (*n* = 268), we prioritized an exploratory approach, and future studies with larger samples should confirm the unidimensional structure using CFA.

## 5. Conclusions

In summary, the present study achieved its objectives by translating and cross-culturally adapting the SFQ to the Brazilian context and assembling a consistent set of psychometric evidence supporting its validity and reliability for measuring the severity of fear of storms in a sample exposed to an extreme weather event. It should be emphasized that although the SFQ is designed for dimensional use and for screening and monitoring purposes, individuals with high scores suggestive of clinically significant impairment should undergo a more comprehensive diagnostic assessment, considering the DSM criteria for storm-specific phobia, to differentiate intense situational fear from an established phobic condition and to guide appropriate therapeutic management. Validation of a suggestive cutoff point for the instrument’s total score, to improve its clinical and epidemiological utility in Brazil, is necessary in future studies. Thus, at this stage, the Brazilian Portuguese SFQ can be used as a standardized measure of storm-related fear severity to support screening and research, facilitate monitoring of symptom burden in affected populations, and enable comparability across studies, while thresholds for clinical decision-making should be established in future work.

## Figures and Tables

**Table 1:** Content validity results.

Item	Mean (Standard Deviation)	CVI
Item 1	3.70 (0.48)	1.00
Item 2	3.50 (1.27)	0.90
Item 3	3.90 (0.32)	1.00
Item 4	3.80 (0.42)	1.00
Item 5	3.90 (0.32)	1.00
Item 6	3.80 (0.42)	1.00
Item 7	3.50 (0.97)	0.90
Item 8	3.80 (0.63)	0.90
Item 9	3.70 (0.48)	1.00
Item 10	3.60 (1.26)	0.90
Item 11	3.90 (0.32)	1.00
Item 12	3.90 (0.32)	1.00
Item 13	3.90 (0.32)	1.00
Item 14	4.00 (0.00)	1.00
Item 15	3.40 (1.35)	0.80

Legend. CVI = Content Validity Index; mean and standard deviation values refer to clarity responses on a 1-to-4 scale. A CVI of 0.80 or higher was considered acceptable.

**Table 2:** Sociodemographic data of the sample.

	Total (*n* = 268)	A (*n* = 63) ^1^	NA (*n* = 205) ^1^	
	Mean (SD)	Mean (SD)	Mean (SD)	*p*-Value
Age	27.67 (6.15)	27.10 (5.91)	27.89 (6.21)	0.375
Previous pregnancies	1.26 (1.41)	1.32 (1.45)	1.26 (1.41)	0.076
Income ^2^	2.5 (1.13)	2.28 (1.12)	2.57 (1.12)	0.102
Educational level ^3^	3.91 (1.76)	3.58 (1.86)	4.01 (1.12)	0.757

Legend: ^1^ A = affected; NA = not affected. ^2^ Interpretation of income data: 1 = less than R$ 1412 (up to one Brazilian minimum wage); 2 = between R$ 1412 and R$ 2999; 3 = between R$ 3000 and R$ 4999; 4 = between R$ 5000 and R$ 9999; and 5 = R$ 10,000 or more. ^3^ Interpretation of educational level data: 1 = incomplete elementary school; 2 = complete elementary school; 3 = incomplete high school; 4 = complete high school; 5 = technical education; 6 = incomplete higher education; 7 = complete higher education. Group comparisons were performed using Student’s *t*-test for independent samples.

**Table 3:** Pearson correlation between the SFQ and the PCL-5, BDI-II, and PES-Brief (*n* = 268).

Variables	SFQ	PCL-5	BDI-II	PES-Brief (Positives)	PES-Brief (Negatives)
PCL-5	0.616 ***	—			
BDI-II	0.427 ***	0.557 ***	—		
PES-Brief (Positives)	0.104	−0.045	−0.153 *	—	
PES-Brief (Negatives)	0.267 ***	0.383 ***	0.461 ***	−0.032	—

Legend: SFQ = Storm Fear Questionnaire; PCL-5 = Posttraumatic Stress Disorder Checklist for DSM-5; BDI-II = Beck Depression Inventory-II; PES-Brief = Pregnancy Experience Scale—Brief. * *p* < 0.05, *** *p* < 0.001.

**Table 4:** Data from the instruments used for the total sample and for the groups.

	Total (*n* = 268)	A (*n* = 63)	NA (*n* = 205)		
	Mean (SD)	Mean (SD)	Mean (SD)	*p*-Value	Cohen’s *d*
SFQ	21.94 (13.97)	25.49 (14.68)	20.86 (13.56)	0.021	0.33
PCL	13.86 (12.73)	19.82 (16.8)	11.98 (10.58)	0.001	0.64
BDI	12.64 (10.96)	14.25 (13.21)	12.20 (10.17)	0.290	0.19
PES+	24.86 (4.32)	25.04 (4.88)	24.81 (4.14)	0.741	0.05
PES−	16.44 (6.53)	16.5 (6.9)	16.4 (6.42)	0.856	0.02

Legend: A = affected; NA = not affected; SD = standard deviation. Group comparisons were performed using Student’s *t*-test for independent samples.

**Table 5:** Factor loadings (component matrix) of the Storm Fear Questionnaire (SFQ) obtained by principal axis factoring.

Item	Component
1	0.554
2	0.591
3	0.575
4	0.737
5	0.621
6	0.653
7	0.586
8	0.757
9	0.564
10	0.523
11	0.647
12	0.534
13	0.576
14	0.600
15	—

Legend: — indicates loadings below 0.40.

**Table 6:** Correlations, means, and standard deviations of the 15 SFQ items.

Item	1	2	3	4	5	6	7	8	9	10	11	**12**	**13**	**14**	**15**
1	-														
2	0.338 ***														
3	0.293 ***	0.361 ***													
4	0.476 ***	0.392 ***	0.452 ***												
5	0.324 ***	0.450 ***	0.449 ***	0.421 ***											
6	0.356 ***	0.371 ***	0.302 ***	0.488 ***	0.364 ***										
7	0.304 ***	0.334 ***	0.219 ***	0.366 ***	0.264 ***	0.591 ***									
8	0.445 ***	0.370 ***	0.393 ***	0.580 ***	0.486 ***	0.472 ***	0.440 ***								
9	0.267 ***	0.404 ***	0.278 ***	0.279 ***	0.310 ***	0.382 ***	0.431 ***	0.422 ***							
10	0.235 ***	0.256 ***	0.297 ***	0.324 ***	0.256 ***	0.397 ***	0.383 ***	0.373 ***	0.369 ***						
11	0.365 ***	0.347 ***	0.402 ***	0.531 ***	0.371 ***	0.426 ***	0.409 ***	0.482 ***	0.420 ***	0.385 ***					
12	0.290 ***	0.294 ***	0.258 ***	0.393 ***	0.284 ***	0.351 ***	0.327 ***	0.439 ***	0.356 ***	0.343 ***	0.296 ***				
13	0.299 ***	0.305 ***	0.223 ***	0.410 ***	0.202 ***	0.542 ***	0.666 ***	0.394 ***	0.403 ***	0.416 ***	0.451 ***	0.375 ***			
14	0.308 ***	0.395 ***	0.329 ***	0.406 ***	0.366 ***	0.377 ***	0.331 ***	0.527 ***	0.329 ***	0.352 ***	0.304 ***	0.372 ***	0.310 ***		
15	0.089	0.170 **	0.120	0.102	0.052	0.122 *	0.114	0.097	0.256 ***	0.249 ***	0.083	0.159 **	0.181 **	0.139 *	
M	1.4	2.02	2.69	1.87	2.86	0.99	0.52	1.76	1.13	0.84	1.34	1.52	0.48	2.28	0.12
SD	1.61	1.75	1.54	1.6	1.42	1.49	1.15	1.61	1.55	1.48	1.64	1.62	1.15	1.67	0.58

Legend: * *p* < 0.05, ** *p* < 0.01, *** *p* < 0.001. M—mean. SD—standard deviation.

## Data Availability

The data presented in this study are available upon reasonable request from the corresponding author. The data are not publicly available because they are part of an ongoing, larger cohort project with continued follow-up of the participants and their children.

## Supplementary Materials

The following supporting information can be downloaded at: https://www.mdpi.com/article/10.3390/brainsci16030288/s1, Supplementary S1: Storm Fear Questionnaire translation process.

## Abbreviations

The following abbreviations are used in this manuscript:

SFQ	Storm Fear Questionnaire
PCL-5	Posttraumatic Stress Disorder Checklist for DSM-5
BDI-II	Beck Depression Inventory II
PES-Brief	Pregnancy Experience Scale—Brief Version
A.I.	Artificial Intelligence
LLM	Large Language Machine
VRS	Verbal Rating Scale
PTSD	Posttraumatic Stress Disorder
ICF	Informed Consent Form
CVI	Content Validity Index
KMO	Kaiser–Meyer–Olkin
