# Supplementary material for: Translation, Cross-Cultural Adaptation, and Validation of the Storm Fear Questionnaire in Brazilian Pregnant Women Exposed to an Extreme Climate Event
Source: Brain Sci. 2026 Mar 4;16(3):288. doi: 10.3390/brainsci16030288 (PMC13023856; doi:10.3390/brainsci16030288)
Supplement: Supplementary file 1 [file brainsci-16-00288-s001.zip › brainsci-4151578-supplementary.pdf]

Supplementary Material 1

Storm Fear Questionnaire translation process.

Original Item	Portuguese translation	Expert review	Back-translation	Final version
**Storm Fear Questionnaire** **Instructions**: Please rate the extent to which each statement is true for you by circling any number from 0 (not at all true) to 4 (almost always true). There are no right or wrong answers.	**Questionário de Medo de Tempestade** **Instruções**: Por favor, avalie até que ponto cada afirmação é verdadeira para você, circulando qualquer número de 0 (nada verdadeiro) a 4 (quase sempre verdadeiro). Não há respostas certas ou erradas.	**Questionário de Medo de Tempestade** **Instruções**: Avalie o quanto cada afirmação é verdadeira para você, circulando um número de 0 (nada verdadeiro) a 4 (quase sempre verdadeiro). Não existem respostas certas ou erradas.	**Storm Fear Questionnaire** **Instructions**: Rate how true each statement is for you by circling a number from 0 (not at all true) to 4 (almost always true). There are no right or wrong answers.	**Questionário de Medo de Tempestade** **Instruções**: Avalie o quanto cada afirmação é verdadeira para você, circulando um número de 0 (nada verdadeiro) a 4 (quase sempre verdadeiro). Não existem respostas certas ou erradas.
1. I worry about storms more than other people.	1. Eu me preocupo com tempestades mais do que outras pessoas.	1. Eu me preocupo com tempestades mais do que outras pessoas.	1. I worry about storms more than other people.	1. Eu me preocupo com tempestades mais do que outras pessoas.
2. I avoid being in a car during a storm for fear that something bad may happen.	2. Eu evito estar em um carro durante uma tempestade por medo de que algo ruim possa acontecer.	2. Eu evito estar em um carro durante uma tempestade por medo de que algo ruim aconteça.	2. I avoid being in a car during a storm for fear that something bad may happen.	2. Eu evito estar em um carro durante uma tempestade por medo de que algo ruim aconteça.
3. I tend to monitor the weather on the radio, TV, internet or in the newspapers to ensure that I know when a storm is coming.	3. Eu costumo monitorar o clima no rádio, TV, internet ou nos jornais para garantir que eu saiba quando uma tempestade está chegando.	3. Eu costumo monitorar o clima no rádio, TV, internet ou em jornais para garantir que eu saiba quando uma tempestade está se aproximando.	3. I tend to monitor the weather on the radio, TV, internet, or in newspapers to make sure I know when a storm is approaching.	3. Eu costumo monitorar o clima no rádio, TV, internet ou em jornais para garantir que eu saiba quando uma tempestade está se aproximando.
4. I tend to get anxious when I hear about a storm approaching, even when the storm is a few days away.	4. Eu costumo ficar ansioso(a) quando ouço falar de uma tempestade se aproximando, mesmo quando ela está a alguns dias de distância.	4. Fico ansioso(a) quando ouço falar de uma tempestade se aproximando, mesmo quando ela está a alguns dias de distância.	4. I get anxious when I hear about a storm approaching, even when it is still a few days away.	4. Fico ansioso(a) quando ouço falar de uma tempestade se aproximando, mesmo quando ela está a alguns dias de distância.
5. I avoid leaving home during a storm to protect myself from possible harm.	5. Eu evito sair de casa durante uma tempestade para me proteger de possíveis danos.	5. Eu evito sair de casa durante uma tempestade para me proteger de possíveis perigos.	5. I avoid leaving home during a storm to protect myself from possible harm.	5. Eu evito sair de casa durante uma tempestade para me proteger de possíveis perigos.
6. I tend to get so anxious about a storm system approaching that I have a hard time functioning normally (for example, difficulty concentrating or sleeping at night).	6. Eu costumo ficar tão ansioso(a) com um sistema de tempestade se aproximando que tenho dificuldade para funcionar normalmente (por exemplo, dificuldade de concentração ou para dormir à noite).	6. Fico tão ansioso(a) com a aproximação de uma tempestade que tenho dificuldade em funcionar normalmente (por exemplo, dificuldade de concentração ou para dormir à noite).	6. I get so anxious about an approaching storm that I have difficulty functioning normally (for example, trouble concentrating or sleeping at night).	6. Fico tão ansioso(a) com a aproximação de uma tempestade que tenho dificuldade em funcionar normalmente (por exemplo, dificuldade de concentração ou para dormir à noite).

Original Item	Portuguese translation	Expert review	Back-translation	Final version
7. When there is a storm, my anxiety is so high that I question whether I can cope.	7. Quando há uma tempestade, minha ansiedade é tão alta que eu me pergunto se consigo lidar com isso.	7. Quando há uma tempestade, minha ansiedade é tão alta que me pergunto se consigo lidar com isso.	7. When there is a storm, my anxiety is so high that I wonder if I can cope with it.	7. Quando há uma tempestade, minha ansiedade é tão alta que me pergunto se consigo lidar com isso.
8. I feel frightened when I see or hear signs of a storm (for example, dark clouds, heavy rain, wind, thunder, or lightning).	8. Eu me sinto assustado(a) quando vejo ou ouço sinais de uma tempestade (por exemplo, nuvens escuras, chuva forte, vento, trovão ou relâmpago).	8. Sinto medo quando vejo ou ouço sinais de uma tempestade (por exemplo, nuvens escuras, chuva forte, vento, trovão ou relâmpagos).	8. I feel afraid when I see or hear signs of a storm (for example, dark clouds, heavy rain, wind, thunder, or lightning).	8. Sinto medo quando vejo ou ouço sinais de uma tempestade (por exemplo, nuvens escuras, chuva forte, vento, trovão ou relâmpagos).
9. When there is a storm approaching, my fear prevents me from going to school, work, or social events.	9. Quando há uma tempestade se aproximando, meu medo me impede de ir para a escola, trabalho ou eventos sociais.	9. Quando há uma tempestade se aproximando, meu medo me impede de ir à escola, ao trabalho ou a eventos sociais.	9. When there is a storm approaching, my fear prevents me from going to school, work, or social events.	9. Quando há uma tempestade se aproximando, meu medo me impede de ir à escola, ao trabalho ou a eventos sociais.
10. When there is a storm approaching, I tend to seek safety in a specific room (for example, a basement, bathroom or hallway).	10. Quando há uma tempestade se aproximando, eu costumo buscar segurança em um cômodo específico (por exemplo, porão, banheiro ou corredor).	10. Quando uma tempestade está se aproximando, costumo procurar segurança em um cômodo específico (por exemplo, porão, banheiro ou corredor).	10. When a storm is approaching, I usually seek safety in a specific room (for example, basement, bathroom, or hallway).	10. Quando uma tempestade está se aproximando, costumo procurar segurança em um cômodo específico (por exemplo, porão, banheiro ou corredor).
11. I worry about being injured or dying as a result of a storm (for example, being struck by lightning).	11. Eu me preocupo em me machucar ou morrer como resultado de uma tempestade (por exemplo, ser atingido por um raio).	11. Me preocupo em me machucar ou morrer como resultado de uma tempestade (por exemplo, ser atingido por um raio).	11. I worry about getting hurt or dying as a result of a storm (for example, being struck by lightning).	11. Me preocupo em me machucar ou morrer como resultado de uma tempestade (por exemplo, ser atingido por um raio).
12. I try to distract myself (for example, listening to music, watching television or reading) during a storm to reduce my anxiety.	12. Eu tento me distrair (por exemplo, ouvindo música, assistindo televisão ou lendo) durante uma tempestade para reduzir minha ansiedade.	12. Tento me distrair (por exemplo, ouvindo música, assistindo televisão ou lendo) durante uma tempestade para reduzir minha ansiedade.	12. I try to distract myself (for example, listening to music, watching television, or reading) during a storm to reduce my anxiety.	12. Tento me distrair (por exemplo, ouvindo música, assistindo televisão ou lendo) durante uma tempestade para reduzir minha ansiedade.
13. I worry that I am going to be harmed or die because of the physical sensations (for example, pounding heart or dizziness) experienced during a storm.	13. Eu me preocupo que vou me machucar ou morrer por causa das sensações físicas (por exemplo, coração acelerado ou tontura) sentidas durante uma tempestade.	13. Me preocupo que vou ser ferido(a) ou morrer por causa das sensações físicas (por exemplo, coração acelerado ou tontura) que experimento durante uma tempestade.	13. I worry that I will be hurt or die because of the physical sensations (for example, rapid heartbeat or dizziness) I experience during a storm.	13. Me preocupo que vou ser ferido(a) ou morrer por causa das sensações físicas (por exemplo, coração acelerado ou tontura) que experimento durante uma tempestade.

Original Item	Portuguese translation	Expert review	Back-translation	Final version
14. I avoid being near windows or open doors during a storm to ensure my safety.	14. Eu evito ficar perto de janelas ou portas abertas durante uma tempestade para garantir minha segurança.	14. Evito ficar perto de janelas ou portas abertas durante uma tempestade para garantir minha segurança.	14. I avoid being near windows or open doors during a storm to ensure my safety.	14. Evito ficar perto de janelas ou portas abertas durante uma tempestade para garantir minha segurança.
15. I use medication, alcohol or drugs to help me cope during a storm.	15. Eu uso medicação, álcool ou drogas para me ajudar a lidar com uma tempestade.	15. Uso medicação, álcool ou drogas para me ajudar a lidar com uma tempestade.	15. I use medication, alcohol, or drugs to help me cope during a storm.	15. Uso medicação, álcool ou drogas para me ajudar a lidar com uma tempestade.

Prompt (Portuguese):

Faça a tradução em temperatura de 0.0 dos seguintes 15 itens e a introdução (mas crie duas colunas, na qual a coluna da esquerda tenha o item original em inglês e a coluna da direita o item traduzido para o português): Storm Fear Questionnaire Instructions: Please rate the extent to which each statement is true for you by circling any number from 0 (not at all true) to 4 (almost always true). There are no right or wrong answers. 1. I worry about storms more than other people. 2. I avoid being in a car during a storm for fear that something bad may happen. 3. I tend to monitor the weather on the radio, TV, internet or in the newspapers to ensure that I know when a storm is coming. 4. I tend to get anxious when I hear about a storm approaching, even when the storm is a few days away. 5. I avoid leaving home during a storm to protect myself from possible harm. 6. I tend to get so anxious about a storm system ap- proaching that I have a hard time functioning normally (for example, difficulty concentrating or sleeping at night). 7. When there is a storm, my anxiety is so high that I question whether I can cope. 8. I feel frightened when I see or hear signs of a storm (for example, dark clouds, heavy rain, wind, thunder, or lightening). 9. When there is a storm approaching, my fear prevents me from going to school, work, or social events. 10. When there is a storm approaching, I tend to seek safety in a specific room (for example, a basement, bathroom or hallway). 11. I worry about being injured or dying as a result of a storm (for example, being struck by lightning). 12. I try to distract myself (for example, listening to music, watching television or reading) during a storm to reduce my anxiety. 13. I worry that I am going to be harmed or die because of the physical sensations (for example, pounding heart or dizziness) experienced during a storm. 14. I avoid being near windows or open doors during a storm to ensure my safety. 15. I use medication, alcohol or drugs to help me cope during a storm.

Prompt (English):

Translate at a temperature of 0.0 the following 15 items and the introduction (but create two columns, where the left column contains the original item in English and the right column contains the item translated into Portuguese):: Storm Fear Questionnaire Instructions: Please rate the extent to which each statement is true for you by circling any number from 0 (not at all true) to 4 (almost always true). There are no right or wrong answers. 1. I worry about storms more than other people. 2. I avoid being in a car during a storm for fear that something bad may happen. 3. I tend to monitor the weather on the radio, TV, internet or in the newspapers to ensure that I know when a storm is coming. 4. I tend to get anxious when I hear about a storm approaching, even when the storm is a few days away. 5. I avoid leaving home during a storm to protect myself from possible harm. 6. I tend to get so anxious about a storm system ap- proaching that I have a hard time functioning normally (for example, difficulty concentrating or sleeping at night). 7. When there is a storm, my anxiety is so high that I question whether I can cope. 8. I feel frightened when I see or hear signs of a storm (for example, dark clouds, heavy rain, wind, thunder, or lightening). 9. When there is a storm approaching, my fear prevents me from going to school, work, or social events. 10. When there is a storm approaching, I tend to seek safety in a specific room (for example, a basement, bathroom or hallway). 11. I worry about being injured or dying as a result of a storm (for example, being struck by lightning). 12. I try to distract myself (for example, listening to music, watching television or reading) during a storm to reduce my anxiety. 13. I worry that I am going to be harmed or die because of the physical sensations (for example, pounding heart or dizziness) experienced during a storm. 14. I avoid being near windows or open doors during a storm to ensure my safety. 15. I use medication, alcohol or drugs to help me cope during a storm.
